# Quantitative Assessment of Upper Limb Motor Function in Ethiopian Acquired Brain Injured Patients Using a Low-Cost Wearable Sensor

**DOI:** 10.3389/fneur.2019.01323

**Published:** 2019-12-12

**Authors:** Charmayne M. L. Hughes, Moges Baye, Chloe Gordon-Murer, Alexander Louie, Selena Sun, Gashaw Jember Belay, Xiaorong Zhang

**Affiliations:** ^1^Health Equity Institute NeuroTech Lab, San Francisco State University, San Francisco, CA, United States; ^2^Department of Kinesiology, San Francisco State University, San Francisco, CA, United States; ^3^Department of Physiotherapy, University of Gondar, Gondar, Ethiopia; ^4^School of Engineering, San Francisco State University, San Francisco, CA, United States

**Keywords:** stroke, kinematics, sensor, rehabilitation, sub-Saharan Africa

## Abstract

Acquired brain injuries place a significant burden on sub-Saharan African rehabilitation clinicians and health care facilities. While wearable sensors have the potential to alleviate these issues, many are beyond the financial capabilities of the majority of African persons and clinics. To bridge this gap, we have developed a low-cost wrist-worn sensor (the outREACH sensor) capable of accurately measuring upper limb movement kinematics. In this study we evaluated the extent to which the outREACH sensor is sensitive to the hand performing the task (unimpaired, impaired) and level of impairment (mild, moderate) in 14 Ethiopian persons with acquired brain injury (mean age = 51.6 ± 12.2 years, 1 female, 13 male). Participants performed an object manipulation task with both the impaired and the unimpaired limb, and reaching performance was measured using standard kinematic measures (i.e., movement time, spectral arc length, peak velocity, peak acceleration, mean velocity, mean acceleration). Overall, movements were smoother and faster when performed by the patient's unimpaired limb. In contrast, maximum velocity did not differ between the two limbs. Moreover, the outREACH sensor was sensitive to differences in performance-based upper limb impairment. Fugl-Meyer assessment for upper extremity scores were significantly correlated with movement time, spectral arc length, and peak velocity. Upper limb movement kinematics can be accurately measured using the outREACH sensor. The outREACH sensor can be a valuable addition to standardized clinical measures that provides rehabilitation clinicians with information regarding initial upper limb impairment level and changes in function across the rehabilitation lifespan.

## Introduction

Each year, acquired brain injuries as stroke and traumatic brain injury, affect millions of persons worldwide (1). The global incidence of these neurological disorders occurs at much higher rates in low- and middle-income countries than in developed countries (2, 3). For example, approximately one-third of all sub-Saharan African patients with traumatic brain injury suffer poor outcomes (4, 5), with severe head injury patients exhibiting twice the risk of dying compared to counterparts from developed countries (4). With regards to cerebrovascular accidents, stroke survivors from sub-Saharan Africa exhibit poorer prognoses (6) and more severe long-term physical disabilities (e.g., weakness or paralysis, sensory loss, immobility, spasticity, and pain) than individuals from developed countries (7, 8).

Patients who survive severe traumatic brain injury or stroke often exhibit upper limb sensorimotor disabilities that negatively influence their ability to perform activities of daily living and have a detrimental effect on patients' capacity for independent living and economic self-sufficiency (9–11). While there is strong evidence that high-intensity task-specific rehabilitation facilitates neural reorganization and motor recovery (12), conventional physical therapy places a significant burden on sub-Saharan health systems, due to the shortage of healthcare professionals and technical resources crucial to the delivery of physical rehabilitation services (13, 14).

The issues surrounding traditional upper limb physical rehabilitation has stimulated particular interest in using wearable sensors for the evaluation post-acquired brain injured upper limb dysfunction (15–18). For example, 12 examined the relationship between stroke impairment level (as measured by the Fugl-Meyer assessment for upper extremity [uFMA] scores) and upper limb kinematics using a 17-sensor system (MVN, Xsens Technologies). Results of that study demonstrated that uFMA scores of stroke patients were positively correlated with maximum reaching distance (*r*^2^ = 0.77), vertical hand elevation (*r*^2^ = 0.7), and reach envelope size (*r*^2^ = 0.7), indicating that wearable sensors are capable of measuring upper extremity motor function and are sensitive to different stroke impairment levels.

Although the extant literature indicates that instrumented wearable technologies are capable of providing sensitive and detailed quantitative information regarding post-acquired brain injury upper limb function, the cost of these devices often exceed the financial capability of persons living in many regions of sub-Saharan Africa. To bridge this gap, San Francisco State University (USA), and the University of Gondar (Ethiopia) have collaborated in order to design a low-cost wearable sensor specifically for use in geographical areas such as Ethiopia, that struggle with a shortage of healthcare professionals and technical resources crucial to the evaluation and rehabilitation of post-acquired brain injured upper limb dysfunction. The validity of the outREACH sensor was recently compared to a Vicon motion capture system (15), with results indicating strong positive correlations (*r* = 0.808–0.990) and agreement (mean difference range: −1.60–1.10) with the reference system. Given that the upper limb movement kinematics of neurologically and physically healthy individuals can be accurately measured, the aim of the present study is to evaluate the extent to which the outREACH sensor is sensitive to the hand performing the task (unimpaired, impaired) and level of impairment (mild impairment, moderate impairment) in 14 Ethiopian acquired brain injury patients with upper limb dysfunction.

## Methods

### Participants

Two traumatic brain injured patients (2 males, mean age = 43.5 ± 29.0, mean time since injury: 18.5 ± 27.7 months) and twelve subacute and chronic stroke patients (11 males, mean age = 53.0 ± 9.3, mean time since injury: 28.3 ± 23.6 months) participated in the present study (Table 1). Study inclusion criteria were shoulder abduction and elbow flexion greater or equal to 3/5 on the Medical Research Council scale for muscle strength, and a Fugl-Meyer Upper Extremity Motor Assessment (uFMA) score of 20–55 or predominant motor ataxia or incoordination (FMA > 55). Participants were excluded if they had any non-stroke or brain injury related arm impairment, moderate arm spasticity as indicated by the Modified Ashworth Scale (>2), moderate shoulder pain (VAS>5/10), visual impairment (hemianopia), visual-spatial neglect, and/or cognitive impairments (Mini Mental State Exam (MMSE) < 26/30). The experiment was approved by the University of Gondar Institutional Review Board (IRB) and was conducted in accordance with the declaration of Helsinki.

**Table 1:** Demographic and clinical characteristics of 14 acquired brain injury (ABI) patients (Type: ICH, intracerebral hemorrhage; IS, ischemic stroke; TBI, traumatic brain injury. Impaired arm: r, Right; L, Left).

**Subject**	**Age**	**Gender**	**ABI type**	**Time since injury (months)**	**Impaired arm**	**FMA**
G001	52	Male	IS	2	Left	36
G002	53	Male	ICH	18	Left	40
G003	42	Male	IS	12	Left	55
G004	73	Male	IS	67	Right	25
G005	62	Male	IS^*^	34	Left	50
G006	53	Male	IS^†^	24	Left	45
G013	48	Female	ICH	2	Left	50
G015	60	Male	ICH	24	Right	53
G016	50	Male	IS	60	Left	47
G028	53	Male	TIA	1	Right	53
G018	37	Male	IS	36	Right	30
G019	53	Male	IS^†^	60	Right	40
G020	23	Male	TBI	36	Right	35
G021	64	Male	TBI	1	Left	55

* *prior stroke*.

† *motor ataxia*.

### Clinical Assessment

The uFMA (19) was used to evaluate sensorimotor function of the upper limb, where a maximum score of 66 on the uFMA indicates normal arm function. It has been shown that the uFMA has excellent reliability (20) and high construct validity with other clinical assessments [i.e., the Action Research Arm Test (21)].

### Kinematic Experimental Equipment and Procedure

**Figure 1:**
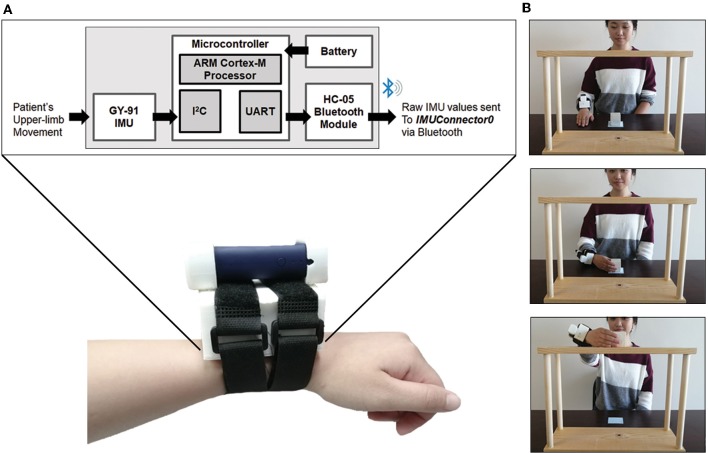
**(A)** Schematic of the outREACH sensor and device placement on a participant's right arm, and **(B)**. The Block Task used to evaluate post-acquired brain injury arm function.

Data was collected from the outREACH custom-built wearable that is 450 g in weight and has a total cost of around $30 USD. As shown in Figure 1A, the sensor consists of a Tiva C Series TM4C123G microcontroller (featuring the ARM Cortex-M4 architecture [Texas Instruments]), GY-91 MPU-9250 Sensor Module, HC-05 Bluetooth module, and a 2,600 mAh USB portable battery (Mophie). The GY-91 MPU-9250 Sensor Module has fully integrated 10-degree-of-freedom measurement capabilities, where the MPU-9250 accelerometer was set for a range of ± 4 g, and the gyroscope was set for a range of ± 500 deg/sec.

After reading and filling out the written informed consent forms, the outREACH sensor was mounted on the dorsal aspect of the wrist (Figure 1B). Participants were seated in a height-adjustable chair in front of the table, so that the center of the sternum was aligned with the center of the shelving unit, and the wrist resting on the tabletop in front of them. Upon the verbal go-signal from the experimenter, the participant reached for the block (5 cm^3^), moved it to the top of the shelf (37.5 cm in height), and then returned their hand to the starting position. Participants performed 20 trials with each hand (impaired, unimpaired), with the order of hand randomized and counterbalanced between participants.

### Kinematic Data Processing and Analysis

At the start of each data collection session, the accelerometer and gyroscope of the GY-91 sensor were recalibrated. Raw sensor frame accelerometer and gyroscope data were read via Inter-Integrated Circuit (I^2^C) communication protocol and sent using Universal Asynchronous Receiver/Transmitter (UART) communication protocol on a HC-05 Bluetooth module to customized recording software at 100 Hz. Data were processed using a custom written MATLAB script. If needed, the raw time-series was trimmed to eliminate early movement performed by the patient.

Next, time periods that the GY-91 sensor was stationary were determined by passing the raw resultant acceleration through a 0.001 Hz High-Pass Butterworth Filter, with the absolute value of the output passed through a 2 Hz Low-Pass Butterworth Filter. The subsequent output of the Low-Pass Filter was then subject to a stationary threshold value as follows. First, the initial threshold was defined as the lowest possible value between 0.025 and 0.1 inclusive such that the first 10 frames or the last 20 frames were less than the threshold. Second, any frames throughout the complete time series below the determined threshold were initially considered to be stationary, with the first 5 frames and the last frame forced to be considered stationary. Third, the first non-stationary and last non-stationary frames were identified, and new thresholding was performed. Last, the time-series between these two frames was subject to a threshold value of 0.018 (determined by manual tuning) and any frames with value less than to 0.018 were considered stationary while frames greater than or equal to 0.018 were consider non-stationary.

The stationary periods, along with the raw sensor frame accelerometer and gyroscope data were passed through an attitude heading reference system (AHRS) to compute orientation and represent the accelerometer and gyroscope data in the earth frame (22). Gravitational acceleration effects were then removed, after which velocity was calculated by taking the integral of acceleration during non-stationary periods. Velocity during stationary periods was forced to be zero. Drift in velocity was calculated and removed from non-stationary periods.

Velocity peak locations were determined by passing the resultant velocity through a peak detect function using variable thresholds. Given the nature of the task (i.e., reach-place-return), the threshold for a specific trial was determined to be the highest threshold for which there were three peaks detected. In the rare case where three peaks were not detected, the threshold was set highest value for the highest number of peaks detected. The time-series velocities and accelerations were trimmed from onset and offset. Onset was defined as the last instance prior to the first peak in which the resultant velocity is <1% of the peak velocity and the mean of the 50 prior frames were <0.1 m/s. The offset was defined as the first instance after the last peak in which the resultant velocity was <0.005 m/s and the mean of the following 75 frames were <0.02 m/s, as these values are indicative of stationary movement.

After segmentation of IMU signals, the following kinematics variables were calculated: total movement time (the time period from movement onset to movement offset), spectral arc length [a dimensionless measure of the arc length of the Fourier magnitude spectrum of the velocity signal, see (23) for more details], mean velocity (average of the resultant velocity signal), peak velocity (highest point on the resultant velocity curve), mean acceleration (average of the resultant acceleration signal), and peak acceleration (highest point on the resultant acceleration curve).

### Kinematic Statistical Analysis

Due to the small sample size, quantitative variables extracted from the outREACH sensor are summarized using descriptive and non-parametric statistics. In particular, differences between hand (impaired, unimpaired) were analyzed with Wilcoxon Signed-Rank Test (24). In addition, Pearson product-moment correlation coefficients were calculated to determine potential associations between reaching and placing kinematics and upper extremity function, as measured by the uFMA (19).

### Semi-structured Interviews

When designing wearable sensor technologies it is imperative to understand the needs of potential users, especially when they are unfamiliar with wearable devices. Indeed, it has been found that low uptake of health technologies in sub-Saharan Africa occurs when product design teams do not take into account the context and user preferences of the community (25). As such, immediately after participants had completed the upper limb object manipulation task, semi-structured interviews were conducted in order to gain an in-depth understanding of barriers and enablers of tele-assessment and rehabilitation from the perspective of Ethiopian patients and family members. The semi-structured interview guide contained initial questions that sought to elicit participant interpretations and awareness of tele-assessment and rehabilitation. These questions helped to orient participants to the topic and guided subsequent questions focused on barriers to, and enablers of, tele-rehabilitation. To aid trustworthiness of data collection, transcript accuracy was checked against interview audio recordings, and the project staff critically reflected on their assumptions, beliefs, and values, and the impact of these on the research process. Thematic analysis of interview data was undertaken following the framework model (26), with themes and deviant cases sought out and examined. Coding was manually performed by research staff, and peer checking was employed to aid credibility and confirmability of data analysis (26), whereby two transcripts were open-coded by a second research staff member. Differences in coding or interpretation of the thematic framework were resolved by discussion between the authors.

## Results

### Movement Kinematics

**Figure 2:**
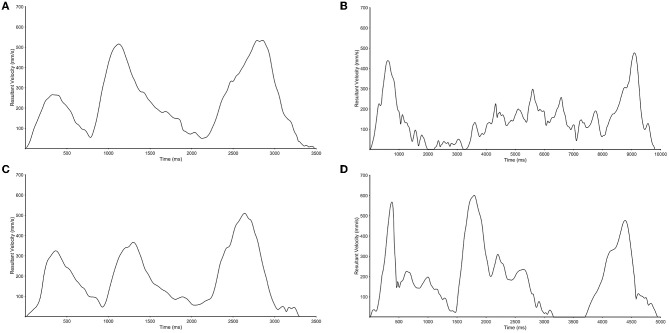
Representative movement trajectories of the unimpaired **(A,C)** and impaired limbs **(B,D)** for a moderately impaired **(A,B)** and mildly impaired participant **(C,D)**.

As shown in Table 2, there were significant differences in reaching performance between the impaired and unimpaired limbs for all three kinematic parameters. In general, movements of the impaired limb were longer and more jerky (Figures 2B,D) than movements performed by the unimpaired limb, which were smooth and featured hand tangential velocity profiles that were bell shaped during each movement segment (Figures 2A,C). Statistical analysis confirmed this observable difference in kinematics. Overall, participants took longer to complete the task with their impaired (5909.66 ms) compared to the unimpaired limb (3293.97 ms), *p* < 0.001. Similarly, movements were smoother when performed by the unimpaired (−2.47) than the impaired limb (−2.82), *p* < 0.001. There were also significant intra-limb differences for the variables mean velocity (impaired = 0.239 m/s, unimpaired = 0.268 m/s) and mean acceleration (impaired = 1.419 m/s^2^, unimpaired = 1.947 m/s^2^), both *p*'s < 0.05. In contrast, the difference between the impaired and unimpaired limb were not significantly different for peak velocity (impaired = 0.652 m/s, unimpaired = 0.268 m/s) and peak acceleration (impaired 7.577 m/s^2^, unimpaired = 7.218 m/s^2^), *p* > 0.05.

**Table 2:** Means, standard deviations for impaired, and impaired upper limbs for the three kinematic parameters.

	**Impaired**	**Unimpaired**	***p*-value**
Movement time (ms)	5909.66 (459.77)	3293.97 (189.67)	0.001^*^
Spectral arc length	−2.816 (1.01)	−2.471 (0.73)	0.001^*^
Peak velocity (m/s)	0.652 (0.20)	0.683 (0.29)	0.122
Mean velocity (m/s)	0.239 (0.12)	0.268 (0.08)	0.011^*^
Peak acceleration (m/s^2^)	7.577 (2.5)	7.218 (2.2)	0.527
Mean acceleration (m/s^2^)	1.419 (0.22)	1.947 (0.45)	0.011^*^

* *p < 0.05*.

Correlations between the outREACH sensor kinematics and performance-based upper limb impairment revealed a number of significant associations. uFMA scores were significantly correlated with movement time (*r* = −0.443), spectral arc length (*r* = 0.360), mean velocity (*r* = 0.390), and peak velocity (*r* = 0.344). In contrast, only low correlations between upper limb impairment and the acceleration variables were observed (peak acceleration *r* = 0.140, mean acceleration *r* = 0.261).

### Semi-structured Interviews

Results of the semi-structured interviews with patients and their caregivers provided valuable insights into the barriers and enablers associated with upper limb tele-assessment and rehabilitation in Ethiopia. Overall, respondents felt there are currently a number of barriers that make tele-assessment and rehabilitation challenging, but that the sensor would decrease these barriers by allowing them to continue physical rehabilitation in the home environment while still being able to communicate with rehabilitation clinicians. As seen in Table 3 the main barriers regarding clinic-based post-acquired brain injury care that patients and caregivers reported were travel time, caregiver burden, and travel and rehabilitation service costs. With respect to time limitations, many participants mentioned that the time required to travel to the hospital was a substantial barrier that influenced how often they went to out-patient physiotherapy. This was an unexpected barrier, given that the majority of participants in our sample lived in Gondar or the surrounding area (i.e., <20 min commute, see Table 2). However, this is a barrier that must not be considered lightly, given that the University of Gondar serves a population of more than five million persons, who come from distances as far away as 196 km).

**Table 3:** Life situations of patients.

**Subject**	**Occupation**	**Education**	**Distance from Hospital (mins)**	**Income (USD month)**	**Smartphone access**	**Sensor affordability**
G001	Hotel manager	12th grade	10	$51	Y	$20.00
G002	Teacher	MSc	10	$272	Y	Cannot afford
G003	Farm Investor	5th grade	5	$7074	Children	$20.00
G004	Retired (Former driver)	6th grade	10	$0	Son	Cannot afford
G005	Retired	None	10	$0	O	Cannot afford
G006	Merchant	4th grade	120	$120	O	$30.00
G013	Housewife	4th grade	15	$102	Son	$34.00
G015	Farmer/Merchant	3rd grade	120	$80	N	Cannot afford
G016	Accountant	Diploma	20	$136	Renter	$20.00
G028	Driver	12th grade	10	$170	Children	$34.00
G018	Accountant	12th grade	18	$119	O	$34.00
G019	English teacher	BSc	30	$136	N	$3.50
G020	Student	9th grade	10	$50	Y	$26.00
G021	Retired soldier	None	10	$38	Daughter	Cannot afford

A second barrier that was mentioned by almost every participant was travel and healthcare service costs. The majority of patients and caregivers stated that having to pay the cost for clinic-based post-acquired brain injury care (0.68USD per session) was financially burdensome. This is unfortunate given that international recommendations are that stroke patients should receive 45 min per day at least 5 days a week, with more rehabilitation added as needed at later stages of recovery (UK, 2012). Additionally, transportation cost was also mentioned as a barrier, with participants paying between 0.14–3.80USD round trip each hospital visit. While these absolute costs may appear to be quite low, one must consider that the median income for the participants is 111.00USD each month (range 0–7,074USD monthly), and that between 80 and 85% of individuals in Ethiopia live on <0.50USD per day^1^. As such, it is not surprising that travel and rehabilitation costs were mentioned as a common barrier.

The last main barrier that emerged from the semi-structured interviews was caregiver burden. All but one patient was accompanied by one or more caregivers whom had to forgo work in favor of bringing them to the hospital in order to receive clinic-based post-acquired brain injury care. Indeed, one patient had to be accompanied by four caregivers who carried them on a stretcher from their village in the mountains to the main road (60-min duration) and then waited on the main road for a taxi to take them to the hospital (60-min duration). The burden of caregivers can thus lead to a loss of wages vital to the family's income, or even job loss.

Additionally, participants also expounded upon the extant barriers likely to influence the implementation of tele-assessment and tele-rehabilitation in the Ethiopian context, namely smartphone accessibility and digital literacy, and sensor cost. Of the 14 participants, only three have smartphones of their own and use it on a regular basis. Nine participants mentioned that they have access to a smartphone through their family or neighbor, but do not have the requisite digital literacy to use the phone without assistance. Despite these challenges, one caregiver stated, “*I am willing to learn how to use Facebook or WhatsApp if it would make distance rehabilitation possible*,” illustrating the motivation and desire of individuals to try new technologies that would decrease the barriers to tele-assessment and rehabilitation. The other main barrier was sensor cost. Of the 14 participants, five could not afford to purchase the sensor at any cost, but of the individuals who could pay, the range of manageable costs was between 3.50–34.00USD (mean = $24.61). Indeed, one participant remarked “*I would like to use the sensor…but with my income I could not afford it*” while another stated “*I cannot afford the sensor and would not pay for it because currently the government pays for my rehabilitation services*.”

## Discussion

In this study, we evaluated the ability of the outREACH sensor to detect differences in upper limb kinematics in Ethiopian acquired brain injury patients. Our results show that the outREACH sensor is sensitive to the hand performing the task (unimpaired, impaired) and level of impairment (mild impairment, moderate impairment) in 14 Ethiopian patients with acquired brain injury. The results of this study indicate that the outREACH sensor is able to accurately measure upper limb kinematics of acquired brain injury patients. In general, movements performed by the unimpaired limb were smooth and featured hand tangential velocity profiles that were bell shaped during each movement segment, whereas movements of the impaired limb were longer and less smooth. These findings are congruent with prior research (27) that demonstrated that the kinematic variables total movement time, smoothness of movement, and movement velocity are strongly influenced by neurological insult, and can be measured by motion capture systems, as well as wearable sensors.

We also found that uFMA scores were significantly correlated with the majority of kinematic variables (i.e., movement time, spectral arc length, mean velocity, and peak velocity), with moderately impaired brain injured patients taking longer to perform the task, and in a less smooth fashion, than mildly impaired patients. These results provide evidence that the outREACH sensor is capable of discriminating between movements performed by the impaired and unimpaired limb of acquired brain injury patients, as well as between different arm impairment levels. Although more data on a larger number of patients is warranted, the relations between overall uFMA score and kinematics indicate that the outREACH sensor provides insight into upper limb motor function that can complement standard clinical assessments. Using these complementary methodologies are likely to improve the assessment of upper-limb dysfunction after acquired brain injury in Ethiopia where there is approximately one physiotherapist per 300,000 persons (28).

Results of the semi-structured interviews also revealed a number of barriers that must be considered as the project moves forward. First, the cost of the outREACH sensor is beyond the financial capabilities of many patients in our sample, as well as the 80–85% of Ethiopian persons who live on < US$0.50 per day^2^. Given the unlikeliness that more cost efficient components will reduce the overall sensor to a price that Ethiopian acquired brain injury patients can afford, we have had to consider alternative business models that are more likely to yield long-term sustainability and scalability of the outREACH sensor. In addition to developing partnerships with Ethiopian stakeholders (e.g., governments, donors, industry), we will utilize a social entrepreneurial model in which Ethiopian and US based for-profit physical rehabilitation clinics would pay a monthly subscription to use the outREACH sensor, and the revenue derived from this market would be used to subsidize costs for resource constrained individuals in Ethiopia.

Second, although patients have access to a smartphone, we need to consider that many future users will have low literacy levels and nascent technology skills. With this in mind, future work will employ a user-centered approach to design a simple, clear, and culturally relevant mobile application available in both English and Amharic (official language of Ethiopia). In addition, we will work to refine the algorithms and integrate them with the outREACH mobile application (15, 29–31) so that upper limb kinematics can be characterized on-line. Despite the numerous barriers for individuals with acquired brain injury, participants provided overall positive feedback regarding the possibility to continue post-acquired brain injury care in their home environment with the outREACH sensor. This is exemplified by a comment made by a caregiver of one participant: “*Distance rehabilitation would be helpful because it can help save time and money which currently make it hard for my family to bring my grandmother to the hospital*.”

Despite the many positive findings, this study includes several limitations that need to be addressed. First, data was collected for a single upper limb reaching, grasping and placing task. While this task was initially selected because of its common usage in post-stroke upper limb function evaluation, the task procedure (i.e., placing a 5 cm^3^ block on a 37.5 cm shelf) restricted testing to patients with mild and moderate acquired brain injury. As such, participants with severe impairments were excluded from participation. We are currently collecting data from tasks in the Graded Repetitive Arm Supplementary Program (i.e., GRASP, e.g., block towers, hanging up the clothes), as this home-based rehabilitation program leads to improvements in paretic limb use during activities of daily living, the ability to perform reaching and grasp movements, and increasing the use of the paretic limb outside of therapy (32). This work will allow us to further investigate the outREACH sensor's ability to detect post-acquired brain injury upper limb impairments in participants with mild, moderate and severe impairments, and to examine relationships between clinical hand function measures (i.e., as measured by the FM-UE wrist and hand subsections) and sensor kinematic measures.

Second, the outREACH sensor was tested in a small group (*n* = 14) of acquired brain injury patients with diverse neurological conditions (i.e., 2 traumatic brain injured patients, 8 ischemic stroke patients, 3 intracerebral hemorrhagic stroke patients, 1 transient ischemic stroke patient) which limits our ability to generalize our findings. However, results of the current study are encouraging, and we will continue this line of research by conducting additional studies with a larger number of patients across a broader neurological profile (e.g., cerebral, right and left hemispheres; lacunar and brain stem), age spectrum, and gender in order to fully evaluate the sensitivity of the outREACH sensor in acquired brain injury patients.

Last, although the collection of data from both impaired and unimpaired limbs allowed us to directly compare healthy and impaired motor performance on an individual basis, the current study provides only a snapshot at a single point in time. Therefore, a longitudinal study would allow us to conclusively state whether this senor is sensitive to acquired brain injury patient's motor improvements over time, a sensitive descriptor of clinical progress.

## Conclusion

Traditional out-patient post-acquired brain injury evaluation and rehabilitation therapy in sub-Saharan Africa is delivered in hospitals located in an urban area, impacting the ability for patients with limited access to rehabilitation clinics to receive adequate rehabilitation care. Leveraging information and communication technologies, as well as IMU technology, we developed a low-cost wearable sensor that would improve the evaluation of post-acquired brain injury upper limb function and may allow patients to continue their rehabilitation program in the home environment. The outREACH sensor can be a valuable addition to standardized clinical measures that provides rehabilitation clinicians with information regarding initial upper limb impairment level and changes in function across the rehabilitation lifespan. The outREACH sensor has the ability to facilitate how physical rehabilitation is delivered in countries such as Ethiopia where a large proportion of the population lives in rural areas and suffer a deficit of experienced health professionals.

## Data Availability Statement

The datasets generated for this study are available on request to the corresponding author.

## Ethics Statement

The studies involving human participants were reviewed and approved by University of Gondar. The patients/participants provided their written informed consent to participate in this study.

## Author Contributions

CH, CG-M, and SS designed the experiment and formulated the experimental question. XZ, CH, AL, and SS developed the sensor. CH, CG-M, MB, GB, and SS collected the data. CH, AL, and SS performed the data analysis and statistics. CH, CG-M, MB, GB, and AL wrote the paper. XZ revised the final version of the manuscript.

### Conflict of Interest

The authors declare that the research was conducted in the absence of any commercial or financial relationships that could be construed as a potential conflict of interest.

## Footnotes

^1^Farm Africa: our work in Ethiopia, https://www.farmafrica.org/us/ethiopia/ethiopia (accessed 5 December 2018).

^2^Farm Africa: our work in Ethiopia, https://www.farmafrica.org/us/ethiopia/ethiopia (accessed 5 December 2018).
